# Cutevariant: a standalone GUI-based desktop application to explore genetic variations from an annotated VCF file

**DOI:** 10.1093/bioadv/vbab028

**Published:** 2021-11-25

**Authors:** Sacha Schutz, Charles Monod-Broca, Lucas Bourneuf, Pierre Marijon, Tristan Montier

**Affiliations:** 1 Univ Brest, Inserm, EFS, UMR 1078, GGB, Brest 29200, France; 2 CHRU Brest, Brest 29200, France; 3 Univ Rennes, Inria, CNRS, IRISA—UMR 6074, Rennes F-35000, France; 4 Heinrich-Heine-Universität Düsseldorf, Medical Faculty, Institute for Medical Biometry and Bioinformatics, Düsseldorf, Germany

## Abstract

**Summary:**

Cutevariant is a graphical user interface (GUI)-based desktop application designed to filter variations from annotated VCF file. The application imports data into a local SQLite database where complex filter queries can be built either from GUI controllers or using a domain-specific language called Variant Query Language. Cutevariant provides more features than existing applications and is fully customizable thanks to a complete plugins architecture.

**Availability and implementation:**

Cutevariant is distributed as a multiplatform client-side software under an open source license and is available at https://github.com/labsquare/cutevariant.

## 1 Introduction

Next-generation sequencing (NGS) has opened new opportunities in genomic research such as identification of DNA variations from Genome, Exome or Panel experiments. In medicine, identification of mutations in rare diseases is a typical use case. These data are delivered as files encoded in the standard Variant Calling Format (VCF version 4.0) where the variations are listed together with the genotype information of different samples. Tools such as VEP (McLaren *et al.*, 2016) or SnpEff (Cingolani *et al.*, 2012a) can be used secondary to add annotations such as genes or functional impact.

Those files are usually the final output yielded by bioinformatic pipelines and are used by end-users to search for mutation of interest. Several management systems have been developed to ease the usage of this filtering step. GEMINI (Paila *et al.*, 2013) and VariantTools (Wang *et al.*, 2014) are command-line applications where data from VCF files are loaded into an SQLite database. Filtering can thus be made very efficiently using the SQL query syntax. Other tools such as SnpSift (Cingolani *et al.*, 2012b) apply filters directly while reading the VCF files line by line, thus avoiding the need to create an intermediate data structure. While these tools are quite flexible, allowing any kind of filtering, the command-line interface is not always accessible for end-users, thus reducing the incentive to use it for non-IT specialists.

This called for the development of applications steered by graphical user interfaces (GUIs). A first approach with this concern in mind was to create web applications. The most popular web applications are private software such as SeqOne (Home, https://seqone.com/), Illumina Base space Interpreter (Variant Interpreter, https://www.illumina.com/informatics/biological-interpretation.html) or Integragen Sirius (SIRIUS, https://integragen.com/fr/bioinformatique/sirius). Some open source solutions exist such as the recently published VarFish (Holtgrewe *et al.*, 2020) or SeqR (Broad Institute, 2021).

A major drawback of this scheme comes from the transit of large amounts of genetic data through public networks raising confidentiality and performance issues, as well as requiring a dedicated server that might not be available for every end-user.

Moreover, these solutions are tailored for human species data and therefore cannot be adapted to projects on other species. A preferable solution is to use a versatile standalone application that can be easily installed on a client computer. VCFMiner (Hart *et al.*, 2016), BrowseVCF (Salatino and Ramraj, 2017) and VCF.Filter (VCF.Filter) implement such a solution. Their main drawback comes from the limited filter settings available through the GUI, since such software lacks domain-specific languages (DSLs). Those are more specific to command-line interfaces.

Despite the availability of these tools, many biologists still use Microsoft Excel to filter their variants and are facing problems such as file size limit, slowness and parsing errors as reported here (Ziemann *et al.*, 2016).

To address the shortcomings of the existing applications, we have developed Cutevariant, a fast GUI-based desktop application implemented in Python that combines both GUI and a DSL called Variant Query Language (VQL) allowing the user to build complex filtering expressions. It is distributed as a multiplatform client-side software under an open source license. Thanks to an architecture based on plugins, Cutevariant is fully customizable, allowing the easy implementation of additional features.

## 2 Methods

**Fig. 1.**
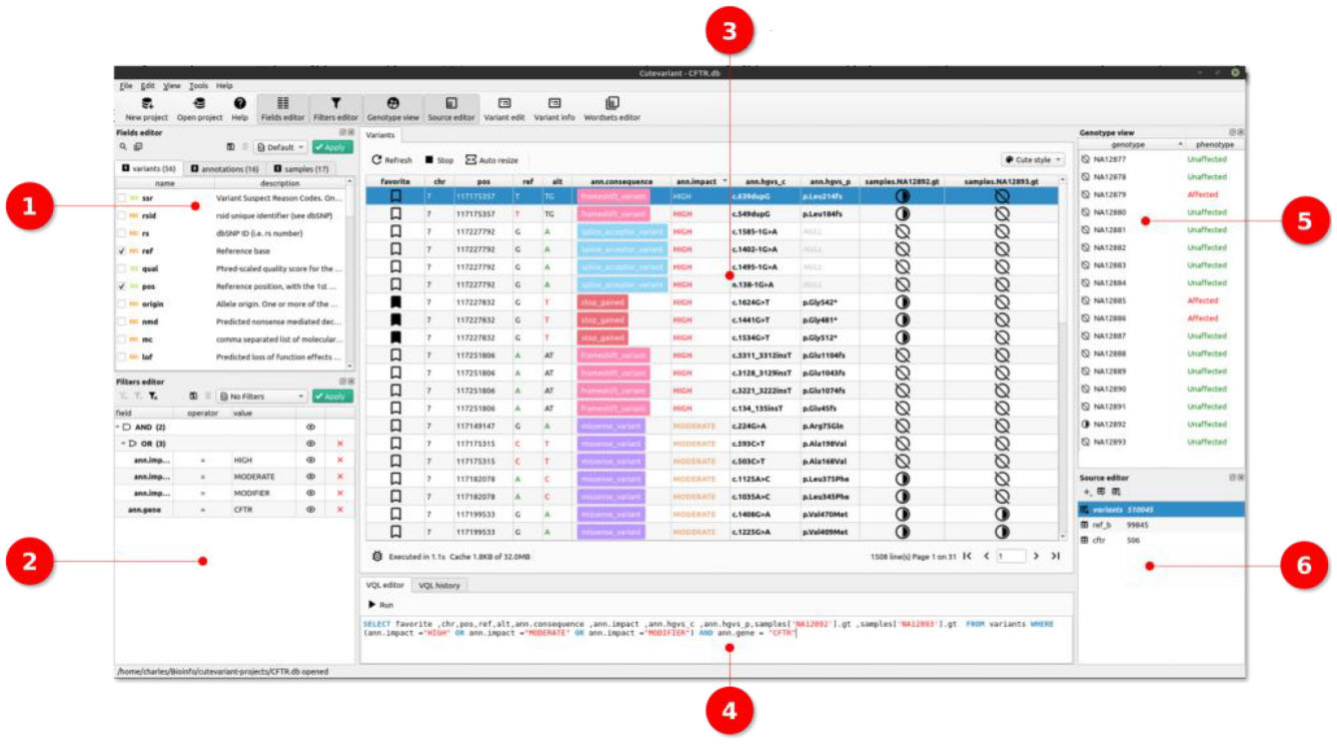
Cutevariant screenshot showing variant view (3) with the VQL editor (4). Different plugins surround the view. The fields editor (1) selects columns. The source editor (6) helps create a subselection of variants. The filter editor (2) creates a nested tree of filter conditions. The genotype view shows, for each sample, their genotype and phenotype for the variant currently selected in the view (3)

Cutevariant is a cross-platform application implemented in Python 3.7 using the Qt5 framework for the user interface (PySide2 ≥ 5.12). Cutevariant imports data from annotated VCF files into an SQLite database. Both SnpEff and VEP are supported. An optional PED file can be provided to describe affected samples and their familial relationship. To facilitate the composition of complex query-filters, the application integrates a DSL named VQL. Grammar was defined using textX (Dejanović *et al.*, 2017) to look like a subset of SQL with specific features for variant filtering. As an example, the following VQL query will select chromosome, position, consequences and genotype of sample NA1223 from variants databases with HIGH impact in the CFTR gene (Fig. 1):

**SELECT** chr, pos, ann.consequence, samples[‘NA1223’].gt **FROM** variants **WHERE** ann.gene = ‘CFTR’ **AND** ann.impact = ‘HIGH’

However, the users do not have to write the VQL queries themselves and can fully rely on the GUI instead. Thanks to a dedicated plugin architecture, one can create interfaces to help the user forge each part of the VQL query. For instance, the filter plugin is intended to build the WHERE clause, while the field plugin controls what columns are shown to the user (the SELECT clause). A more specific plugin is the Trio Analysis that makes it possible to filter variants depending on their transmission mode of inheritance.

It is also possible to change the style of the application using plugins. For instance, from a python script, one can easily change the style of the cell with different colors, text or icons according to the value of the cell.

## 3 Results

### 3.1 Use case: cohort analysis

We have repeated with Cutevariant the analysis given as an example by SnpSift (Examples—SnpEff & SnpSift Documentation, Cingolani *et al*. 2012a). It is a cohort analysis of 17 individuals among which three are affected by a nonsense mutation in the CFTR gene (G542*). This analysis cannot be performed with any of the standalone GUI applications listed previously (Table 1). After importing the annotated VCF file and the corresponding PED file, the following VQL query was processed by Cutevariant to select variants with HIGH impact which are homozygous in case samples but are not in control samples:

**SELECT** chr, pos **FROM** variants **WHERE** case_count_hom=3 **AND** control_count_hom=0 **AND** impact **IN** ('HIGH', ‘MODERATE’)

The SnpSift equivalent provides the same results reads as:

cat protocols/ex1.ann.cc.vcf | java -jar SnpSift.jar filter (Cases[0]=3) & (Controls[0]=0) & ((ANN[*].IMPACT="HIGH")|(ANN[*].IMPACT='MODERATE')) > protocols/ex1.filtered.vcf

**Table 1.** Features available in various applications available on the market

	GUI						Command line		
Features	Cutevariant	BrowseVCF	VCF-Miner	VCF-Explorer	VCF-Server	VCF-Filters	GEMINI	Variant tools	SnpSift
Process annotations	No	No	No	No	Yes	No	Yes	No	No
VEP parser	Yes	Yes	No	No	No	No	Yes	No	No
SnpEff parser	Yes	Yes	No	No	No	No	Yes	Yes	Yes
SQL like query	Yes	No	No	No	No	No	Yes	Yes	Yes
Regular expressions	Yes	No	No	No	No	No	No^a^	No^a^	Yes
Bed file intersection	Yes	No	Yes	No	No	Yes	No	No	Yes
Set operations	Yes	No	No	No	No	No	No	Yes	Yes
Sorting	Yes	Yes	Yes	No	Yes	No	Yes	Yes	Yes
Intersect with wordset	Yes	Yes	No	No	No	No	No	No	Yes
Plugins extension	Yes	No	No	No	No	No	No	No	No
Indexed database	SQLite	Berkeley DB	MongoDB	Raw file	MongoDB	Raw file	SQLite	SQLite	Raw file
data encryption	No^b^	No	No	No	Yes	No	No	No	No
Language	Py3/Qt	Py2/HTML	JS/HTML	C++/Qt	Node.js	Java	Py3	Py3	JAVA
pedigree file	Yes	No	No	No	No	No	Yes	Yes	Yes
Application type	Desktop	Web	Web	Web	Web	Desktop	Console	Console	Console
Multi-users support	No	No	No	No	Yes	No	No	No	No
CSV/Excel export	Yes	Yes	Yes	Yes	Yes	No	Yes	Yes	Yes

a Support LIKE SQL expression.

b Possible with SQLITE encryption extension.

### 3.2 Performance

In Table 2, we compared the timing performance of VCF importation and indexation with VCF-Miner (Hart *et al.*, 2016), the fastest application we evaluated. Cutevariant outperforms VCF-Miner except for 1KG.chr22.anno.vcf. This is because this VCF file contains many samples and cutevariant required here to compute normalized tables.

**Table 2.** Comparison of time performance between cutevariant and VCF-miner for importation/indexation and query execution

File used	Cutevariant	VCF-miner
Import 1KG.chr22.anno.vcf (Data | 1000 Genomes)	6600 s	2940 s
Import corpas.quartlet.vcf (Corpas, 2013)	78 s	183 s
Import NA12878.vcf (Zook *et al.*, 2016)	810 s	2200 s
Query time execution	0.02 s	≈ 1 s

*Notes:* Query time execution was measured to select variants with QUAL ≥30 and DEPTH ≥ 30. Executed on Intel(R) Core(TM) i5-3570K CPU @ 3.40 GHz with 16 Gb RAM.

## 4. Conclusion

Cutevariant is a new desktop application devoted to exploring genetic variations in NGS data. It is the first GUI software that integrates both GUI and a DSL to satisfy both IT and non-IT specialists. It is particularly suitable for biologists to analyze annotated VCF produced upstream by a bioinformatics pipeline.

Thanks to its low learning threshold, end-users can easily perform complex filtering with VQL to identify variants of interest. Cutevariant is also fully customizable thanks to its plugin-based implementation and thus offers features and modularity that are not available with existing applications.

Cutevariant is a standalone application that runs on standard desktop computers either under Linux, MacOS or Windows operating systems. The plugins architecture makes the application easily expandable with the addition of new features, thus offering the possibility to involve the community at large in new features developments.

## Funding

This work was supported by Université de Bretagne Occidentale (UBO), France and CHRU, Brest, France.

*Conflict of Interest:* none declared.
